# Interplay between hepatic mitochondria-associated membranes, lipid metabolism and caveolin-1 in mice

**DOI:** 10.1038/srep27351

**Published:** 2016-06-06

**Authors:** Aleix Sala-Vila, Inmaculada Navarro-Lérida, Miguel Sánchez-Alvarez, Marta Bosch, Carlos Calvo, Juan Antonio López, Enrique Calvo, Charles Ferguson, Marta Giacomello, Annalisa Serafini, Luca Scorrano, José Antonio Enriquez, Jesús Balsinde, Robert G. Parton, Jesús Vázquez, Albert Pol, Miguel A. Del Pozo

**Affiliations:** 1 Centro de Investigación Biomédica en Red de Fisiopatología de la Obesidad y Nutrición, Instituto de Salud Carlos III, Madrid, Spain; 2 Hypertension, lipids and cardiovascular risk Group, Institut d’Investigacions Biomèdiques August Pi Sunyer (IDIBAPS), Barcelona, Spain; 3 Integrin Signaling Laboratory, Cell & Developmental Biology Area, Centro Nacional de Investigaciones Cardiovasculares Carlos III (CNIC), Madrid, Spain; 4 Cell Compartments and Signaling Group, Institut d’Investigacions Biomèdiques August Pi i Sunyer (IDIBAPS), Barcelona, Spain; 5 Cardiovascular Proteomics Laboratory, Centro Nacional de Investigaciones Cardiovasculares Carlos III (CNIC), Madrid, Spain; 6 The Institute for Molecular Bioscience; Centre for Microscopy and Microanalysis, The University of Queensland, Brisbane, Queensland, Australia; 7 Dulbecco Telethon Institute, Venetian Institute of Molecular Medicine, Padova, Italy; 8 Department of Biology, University of Padova, Padova, Italy; 9 Functional Genetics of the Oxidative Phosphorylation System, Centro Nacional de Investigaciones Cardiovasculares Carlos III (CNIC), Madrid, Spain; 10 Instituto de Biología y Genética Molecular, Consejo Superior de Investigaciones Científicas, Universidad de Valladolid, Valladolid, Spain; 11 Centro de Investigación Biomédica en Red de Diabetes y Enfermedades Metabólicas Asociadas, Instituto de Salud Carlos III, Madrid, Spain; 12 Departament de Biologia Cel·lular, Immunologia i Neurociències, Facultat de Medicina, Universitat de Barcelona, Barcelona, Spain; 13 Institució Catalana de Recerca i Estudis Avançats (ICREA), Barcelona, Spain

## Abstract

The mitochondria-associated membrane (MAM) is a specialized subdomain of the endoplasmic reticulum (ER) which acts as an intracellular signaling hub. MAM dysfunction has been related to liver disease. We report a high-throughput mass spectrometry-based proteomics characterization of MAMs from mouse liver, which portrays them as an extremely complex compartment involved in different metabolic processes, including steroid metabolism. Interestingly, we identified caveolin-1 (CAV1) as an integral component of hepatic MAMs, which determine the relative cholesterol content of these ER subdomains. Finally, a detailed comparative proteomics analysis between MAMs from wild type and CAV1-deficient mice suggests that functional CAV1 contributes to the recruitment and regulation of intracellular steroid and lipoprotein metabolism-related processes accrued at MAMs. The potential impact of these novel aspects of CAV1 biology on global cell homeostasis and disease is discussed.

Mitochondria-associated membranes (MAMs) are specialized membrane subdomains that allow for communication between the endoplasmic reticulum (ER) and the mitochondria. MAMs were primarily proposed to constitute areas gating the interchange of inorganic ions and complex lipids between these two organelles- indeed, these physical contacts determine calcium fluxes transducing diverse stimuli such as proapoptotic cues^1^, and allow for specific lipid transactions such as the conversion of ER-derived phosphatidylserine into phosphatidylethanolamine and other phospholipid species on the mitochondrial surface^2^. However, MAMs have subsequently been shown to accrue multiple specific activities and are currently conceived as proactive intracellular hubs, integrating numerous signaling and trafficking pathways, and coordinating the metabolic status of the cell with other cellular processes^3^. Among these processes, the regulated trafficking of cholesterol and its use as a precursor for steroid derivatives is highly represented. Thus, MAMs have the potential to propagate specific functional imbalances to other systems of the cell. Accordingly, increasing evidence links MAM dysfunction with complex diseases such as neurodegenerative disorders, aberrant lysosomal storage syndromes, obesity-related pathologies and diabetes, and cancer^1^,^4^. MAM dysregulation in the liver has been proposed to contribute to the development of insulin resistance and hepatosteatosis^1^. MAMs may also contribute to other major health threats such as hepatitis C infection and hepatocarcinoma, because of the pivotal relevance of aberrant inflammation and proteostasis signaling in these pathologies^3^. Therefore, MAMs may constitute a promising therapeutic target for liver disease. However, our knowledge about this domain is still scarce and the molecular machinery that functionally regulates MAMs has not been fully elucidated yet.

Here we describe an in-depth mass spectrometry characterization of highly purified MAM fractions from mouse liver. Fatty acid catabolism and steroid metabolism appear among the most enriched functional classes annotated for liver MAM components, and we were able to map most key components of the cholesterol/steroid biosynthesis and transport pathways. Intriguingly, we also found caveolin-1 (CAV1), a pivotal regulator of cholesterol intracellular transport and membrane organization, as an specific integral component of MAMs. Because of the relevance of CAV1 for mitochondrial functioning, lipidostasis and metabolic homeostasis^5^,^6^, and for the control of numerous signaling pathways integrated at MAMs^7^, we performed a comparative structural and compositional study between wild type (henceforth WT) and CAV1-deficient mice (CAV1KO). CAV1 genetic deficiency leads to reduced MAM physical extension and aberrant free cholesterol accumulation at these ER subdomains. Quantitative mass spectrometry reveals that specific, functionally coherent regulators are altered, with a particular impact on steroid biosynthesis and inorganic ion transport. The potential relevance and future avenues of research suggested by the present framework are discussed.

## Results

**Figure 1:**
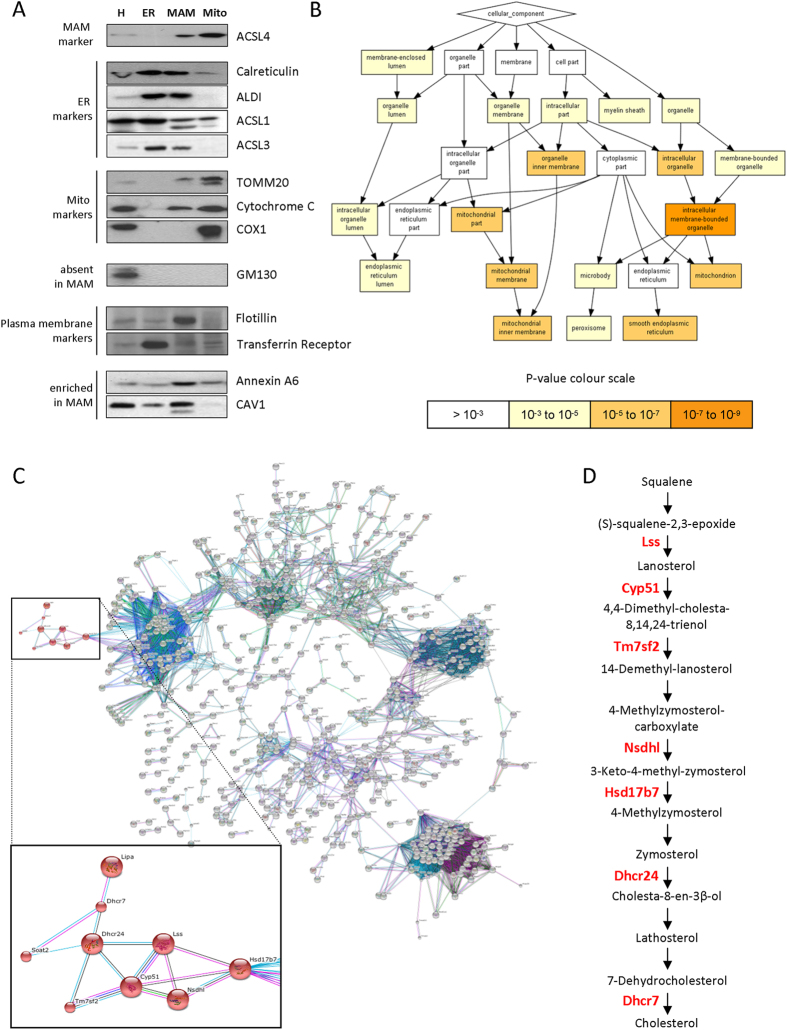
(**A**) Equal total protein amounts of liver homogenates (H), purified endoplasmic reticulum (ER), mitochondria-associated membranes (MAM), and mitochondria (MITO) fractions from C57BL/6 mice, were resolved by SDS-PAGE and analyzed by western blotting for the indicated gene products. Their expected preferential enrichment is indicated by the left handside textbox groupings. (**B**) Hierarchical GOrilla diagram for “cellular component” annotation category enrichment for MAM components identified (n = 3). Colour hue code indicates P value significance (reference below the tree). (**C**) STRING (Search Tool for the Retrieval of Interacting Genes/Proteins) network analysis assessing interactions among identified MAM components. Inset shows in higher detail a cluster comprising steroid biogenesis regulators. (**D**) Mapping of the MAM components (red font) to the KEGG pathway describing the conversion of squalene to cholesterol (module M00101).

We slightly modified current guidelines for the purification and functional analysis of MAMs^8^ obtaining highly purified MAM fractions from healthy, adult mouse livers (Supplementary Fig. 1). We first assessed the purity of our fractions by western blot analysis (Fig. 1A). In agreement with published data, MAMs were highly enriched in acyl-CoA synthetase long chain 4 (ACSL4/FACL4), a well-established MAM marker^8^. ER-resident proteins were differentially distributed: calreticulin and associated with lipid droplet protein 1 (ALDI) were similarly partitioned between ER and MAM fraction, but the acyl-CoA synthases ACSL1 and ACSL3 were highly reduced in MAMs compared to the bulk of the ER. MAM-containing fractions displayed trace amounts of mitochondrial proteins such as TOMM20 (outer membrane) and cytochrome C (intermembrane space), and mitochondrial-inner membrane proteins such as the cytochrome C oxidase subunit 1 (Cox1/Mtco1) were not detectable, supporting a high specificity of the purified MAM membrane fractions. Other membrane-bound compartment markers such as the cis-Golgi protein GM130 were neither detected in our samples. Analysis of plasma membrane markers such as transferrin receptor (liquid-disordered –l_d_-_ domains marker) and flotillin (liquid-ordered –l_o_- domains marker) in MAM fraction revealed absence of the former, while a significant enrichment of the latter. These results confirmed the previously reported l_o_-like nature structure of MAMs^9^ while supporting a low plasma membrane contamination in this purified fraction (Fig. 1A).

Interestingly, two proteins involved in vesicular transport and essential for hepatic metabolism and cholesterol homeostasis, annexin A6 (a Ca^+2^ and phospholipid binding protein) and CAV1 (a cholesterol-binding protein) were also found to be enriched in MAMs (Fig. 1A).

We then performed in-depth mass spectrometry analysis of the proteins comprising MAM-enriched fractions (n = 3 replicates). A list of 1052 proteins was identified in all replicates by at least two unique peptides with a FDR < 1% (Supplemental File 1, sheet A), a much stricter criterion than those typically used in proteomics. Due to poor performance of resulting tryptic peptides and the relative low amount compared to other proteins, CAV1 itself was only detected when immunoprecipitation enrichment was performed previous to MS analysis (Supplemental File 1, sheet B). The analysis of the proteins with GOrilla (http://cbl-gorilla.cs.technion.ac.il), indicated enrichment of smooth endoplasmic reticulum and/or mitochondrial proteins within the ontology “cellular component” (Fig. 1B). Supplemental File 1, sheets C–E summarize the single ranked list, sorted by p-value for ontologies of “cellular component”, “function”, and “process”, respectively. It must be noted that the procedure allows for rather specific enrichment of the MAM subdomains because we did not observe any accompanying enrichment in plasma membrane categories- not even when assessing the functional enrichment of MAM components against a mitochondrial reference database (MitoCarta; data not shown).

To infer potential relationships among the identified MAM protein components, the list of proteins (Supplemental File 1, sheet A) was entered into STRING 9.0 (www.string-db.org). Figure 1C shows a map using the most stringent criteria for physical, genetic and functional interactions. We focused on a cluster comprising nine proteins belonging to the “Steroid biosynthesis” KEGG Pathway (mmu00100) (inset). Figure 1D summarizes the seven MAM-resident proteins involved in the conversion of (S)-squalene,2-3-epoxyde to cholesterol. The cluster also contained a protein involved in intracellular hydrolysis of cholesteryl esters (Lipa), and the major hepatic cholesterol esteryfing enzyme (Soat2).

**Figure 2:**
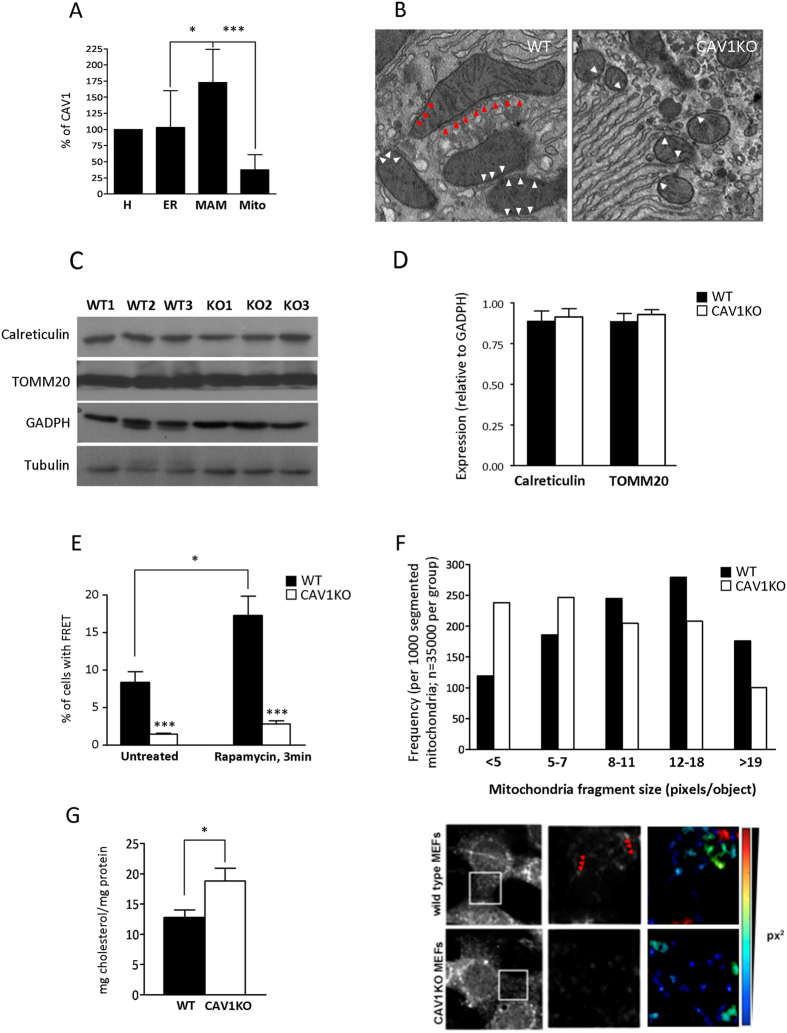
(**A**) Quantification of relative CAV1 levels (normalized to total homogenate) present in purified endoplasmic reticulum (ER), mitochondria-associated membranes (MAM) and mitochondria (MITO). Densitometry data from western blot analysis is derived from three independent fractionation experiments from wild type livers. (**B**) Electron microscopy images of WT and CAV1KO livers. In the CAV1KO the contacts are small, at the ends of tubules or cisternae, while in WT ER cisternae were often observed running along mitochondria making contact over a greater area (white arrows). A characteristic fenestrated-like appearance is observed in the WT but lacking in the CAV1KO (red arrows). (**C**) Western blotting analysis for calreticulin (marker of endoplasmic reticulum) and TOMM20 (marker of mitochondria) in liver homogenates from 3 WT and 3 CAV1KO. GADPH and tubulin were used as a loading control. (**D**) Quantification of calreticulin and TOMM20 relative to GAPDH in liver homogenates from WT and CAV1KO, from samples resolved and immunoblotted in (**C**). (**E**) FRET signal from WT and CAV1KO MEFs transiently expressing the ER-mitochondria tethering pair, in the absence (untreated) or presence (3min) of rapamycin. (**F**) Quantitative image analysis of mitochondria fragmentation. [TOP]: Size distribution of mitochondria fragments in five size classes (see Materials and Methods). [BOTTOM]: Representative spinning disk confocal images of WT and CAV1KO MEFs stained with MitoTracker FarRed. Lower panels show enlarged views of the peripheral mitochondrial network, with red arrowheads indicating elongated mitochondria extensions. Pseudocolored inlays show the raw segmentation mask of individual mitochondrial fragments, reporting the size of the detected fragments in a color scale (bottom right, in square pixels). (**G**) Cholesterol content in isolated-hepatic MAMs from WT and CAV1KO from three independent experiments, measured using a fluorometric assay.

CAV1 is best known as an essential structural component of plasma membrane invaginations (*caveolae*) with proposed roles in signaling, endocytosis and mechanosensing/mechanoprotection^7^. However, CAV1 is assembled onto higher-order complexes in the ER, is required for the regulation of cholesterol efflux, and is required for mitochondrial homeostasis^5^,^6^. Our results demonstrate that CAV1 is specifically enriched in MAM-containing fractions as compared with other intracellular compartments (Fig. 2A). CAV1KO display phenotypes which are currently associated with MAM dysregulation, such as aberrant lipid management and metabolism^10^. A plausible hypothesis is that MAM dysfunction is an underlying causal component of the pathological phenotypes displayed by CAV1-deficient backgrounds. Thus, we decided to assess the impact of CAV1 deficiency on MAM integrity and composition.

First, we studied the physical properties and extension in MAMs from CAV1KO as compared with the WT. Figure 2B shows representative transmission electron microscopy images from liver samples from both WT and CAV1KO^11^. WT exhibited more extensive ER-mitochondria contacts, both in % of mitochondria contacting ER (70.0 ± 11.8% in WT vs. 53.0 ± 13.0% in CAV1KO) and in absolute length (28.1 ± 3.2 nm in WT vs. 11.5 ± 2.6 nm in CAV1KO) (p < 0.05, both). This could not be attributable to a lower mass of mitochondria and/or ER in CAV1KO, given the lack of significant differences in both markers of ER (calreticulin) and mitochondria (TOMM20) normalized to GAPDH and/or tubulin in liver homogenates (n = 3 for each genotype; Fig. 2, panels C,D). Furthermore, total mitochondrial mass estimation by flow cytometry suggests that CAV1KO mouse embryonic fibroblasts (MEFs) from the same genetic background as the animals used for liver sample harvesting have modestly higher total mitochondrial mass than wild type counterparts (Supplementary Fig. 2A). It must be noted nonetheless that superresolution microscopy analysis of both the mitochondrial network and the ER compartment display apparent aberrancies in CAV1-deficient backgrounds, either consequence from or precursory of diminished ER-mitochondria apposition (Supplementary Fig. 2B).

We further assessed the impact of CAV1 deficiency on MAM physical extension and integrity using inducible artificial ER-mitochondrial tethering reporters on MEF cell lines^12^. Briefly, a FRET pair (a CFP-containing, ER-targeted module; and an YFP-containing, outer mitochondrial membrane-targeted module) can be stabilized by the addition of rapamycin owing to their respective homodimerizing FKBP domains. The FRET signal, dependent on the extension of close ER-mitochondria apposition, can then be quantified by flow cytometry as a proxy of MAM extension and stability. We found a significantly lower FRET signal in the CAV1KO cells both in the absence and in the presence of the stabilizing reagent (Fig. 2E), further supporting a reduced MAM extension/stability associated with CAV1 deficiency.

Considering that ER-mitochondria contacts allocate regulators of mitochondrial dynamics and morphology, we decided to explore the involvement of CAV1 deficiency in this process. As shown in Fig. 2F and Supplementary Fig. 2C,D, absence of CAV1 exhibited an increased mitochondria fragmentation pattern in contrast to the nice elongated mitochondrial network displayed when CAV1 is present. Quantitative analysis by using the Opera automated microscopy and the Acapella Studio Software package (see Materials and Methods) rendered a differential average distribution of the mitochondria profile in wild-type and CAV1-deficient cells.

We next studied the impact of CAV1 deficiency on MAM composition. We first subjected isolated MAM fractions from WT and CAV1KO livers (n = 3, each genotype) to free cholesterol analysis. The levels of cholesterol in MAM were significantly increased in the absence of CAV1 (Fig. 2G), in agreement with the role of CAV1 in cholesterol efflux and endomembrane cholesterol level control. Consistent with previously published data^6^, the levels of cholesterol in purified ER and mitochondria were also significantly increased in the absence of CAV1 (ER, 7.2 ± 0.5 vs. 9.5 ± 1.1 mg cholesterol/mg protein, P < 0.01, for WT and CAV1KO, respectively; mitochondria, 4.8 ± 1.5 vs. 6.7 ± 1.7 mg cholesterol/mg protein, P < 0.01, for WT and CAV1KO, respectively). However, purified MAM was significantly enriched in cholesterol when compared to the ER (x1.8 in WT and x2.0 in CAV1KO) and mitochondria (x2.6 in WT and x2.8 in CAV1KO). Interestingly, cholesterol accumulation in MAMs can contribute, but it is not likely the direct cause of, MAM disruption, because reduction of endogenous cholesterol levels by short-term treatment with the HMG-CoA reductase inhibitor lovastatin did not rescue significantly the drop of FRET signal observed in CAV1 KO cells (data not shown).

**Figure 3:**
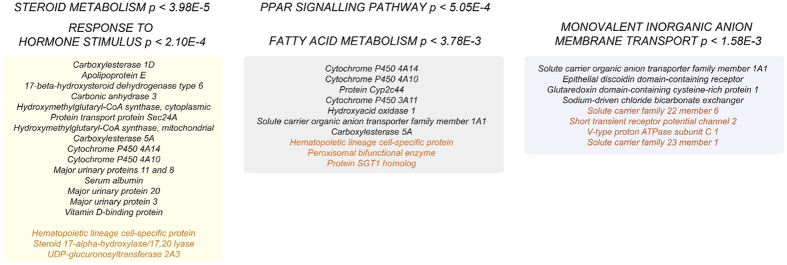
Functional annotation categories enriched for proteins differentially represented in CAV1KO as compared with WT MAMs are depicted in colour hue boxes, listing within the annotated hits from either WT (black font) or CAV1KO backgrounds (orange font).

We then profiled the differential protein compositions of WT versus CAV1KO MAMs (n = 2, each genotype) using multiplexed isobaric labeling and quantitative mass spectrometry analysis. From the total list of quantified proteins we selected those with absolute values of Zq (standardized log2-ratios) ≥1.5; using this criterion, 29 proteins were found to be depleted, and 22 enriched in hepatic MAMs from CAV1KO as compared to WT (Supplementary File 2). Notably, functional annotation categories could be identified when grouping these genes (Fig. 3). Steroid metabolism is the most salient category and key regulators of cholesterol biosynthesis, such as the HMG-CoA synthase (Hmgcs1 and 2), were significantly depleted in MAM fractions from CAV1KO (Fig. 3; Supplementary File 2). Of note, these relative differences are not likely derived from changes in total levels in the tissue, as previous analyses from whole cell lysates have not revealed such an extent of depletion in CAV1KO backgrounds (data not shown), thus suggesting a potential specific role of CAV1 on the sorting and accruing of these functions at MAMs. A potentially relevant specific finding is the relative depletion from CAV1KO derived MAMs of apolipoprotein E, a key structural component of VLDL particles and an essential positive regulator of cholesterol efflux and extracellular transport. Another minor functional category significantly depleted from CAV1KO comprises genes related to fatty acid catabolism and beta-oxidation- the impairment of these processes upon CAV1 genetic ablation has been well documented previously. These findings further stress the essential functional relevance of liver MAMs in energy management and fatty acid catabolism as outlined by our previous in-depth analysis. Conversely, MAM-containing fractions from CAV1KO were significantly enriched in a number of membrane cation transporters- because Ca2+ transport gating is a key function of MAMs upon stimuli such as apoptotic triggering (Fig. 3). These differences might partly explain mechanisms underlying apoptotic regulation and survival displayed by several CAV1KO models *in vitro*.

In summary, we provide a novel and accurate mass spectrometry-based proteomics characterization of the MAMs from mouse liver. Our analysis highlights lipid metabolism, energy management and steroid anabolism as major regulated targets of MAM functions. Furthermore, we describe CAV1 as a novel potential key modulator of both the physical integrity and the function of ER-mitochondria interactions. These findings have implications of relevance, regarding both the complex metabolic phenotypes displayed in caveolinopathies and the physiopathological role and therapeutic potential of CAV1 in multiple complex diseases that potentially course with MAM aberrant functioning, such as diabetes, obesity, hepatosteatosis and cancer.

## Discussion

In this work we describe for the first time the protein composition of highly purified hepatic MAM fractions and demonstrate that CAV1 is a MAM-resident protein. In addition, we describe structural and protein alterations that take place in MAMs when the CAV1 gene is non-functional.

Our unprecedented profiling of hepatic MAMs confirms that several enzymes involved in lipid metabolism (in particular conversion of (S)-squalene,2-3-epoxyde to cholesterol, and biosynthesis of steroid derivatives) accrue at hepatocyte MAMs. Interestingly, we also found a significant enrichment in regulators of energy management by mitochondria and fatty acid catabolism. These findings, in the context of physiological liver functioning, highlight the potential relevance of MAMs as an integrative hub coordinating several processes that are regulated by multiple cues such as growth factor signaling, energy balance, and cell anabolism. For example, insulin signaling is a well-established determinant of MAM extension and integrity, and through this parameter several lipid metabolism functions could be regulated, simultaneously engaging in functional crosstalk with the mitochondria to fine-tune energy management. Our datasets also constitute an excellent framework for comparative studies of the regulation of MAM components across different conditions or pathological models.

Our observations also demonstrate CAV1 as a novel MAM component, as suggested by previous observations^13^,^14^. This further reinforces the notion that besides the critical role in the assembly of *caveolae* (cholesterol-enriched membrane microdomains of most mammalian cells), CAV1 plays additional intracellular functions, particularly in the ER^6^,^7^,^15^. Previous studies demonstrate that CAV1 genetic ablation affects mitochondrial function^5^,^16^ through molecular mechanisms not demonstrated as yet. We report that CAV1 genetic ablation results in a markedly reduced extension/stability of ER-mitochondria contacts. Several facts might explain these observations in a non-mutually exclusive manner. First, an apparent remodeling of ER and mitochondria architecture is displayed by CAV1KO cells. Although MAM disruption might result unspecifically from aberrant ER and/or mitochondrial architecture, it must be noted that at least one of the most prominent traits of CAV1 (mitochondrial fission) is shared by other genetic perturbations that *also* disrupt MAM integrity, such as mitofusin 2 genetic ablation^17^. On the other hand, the metabolic alterations associated with CAV1 deficiency (glucose addiction, OXPHOS upregulation, impairment of fatty acid beta-oxidation) have also been associated themselves with elevated degrees of mitochondrial fragmentation^18^. Undergoing genetic screens may establish the causal relationship among mitochondrial fragmentation, ER architecture and MAM integrity, and the metabolic reprogramming that is associated with CAV1 deficiency.

We hypothesized, in a similar manner as had been observed for core mitochondrial function, that a key mechanistic component of these alterations is likely the observed accumulation of free cholesterol in MAMs of CAV1KO (Fig. 2G). However, treatments that significantly decrease the endogenous pool of cholesterol (i.e. acute lovastatin exposure) do not significantly rescue MAM integrity as assessed by our FRET-based assay. We propose that several functional layers of membrane integrity control are supported by CAV1 regarding MAM composition. First, cholesterol efflux could passively reduce the amount of cholesterol diffusing to different intracellular compartments such as MAMs, mitochondria and ER^5^,^6^. Second, MAM-resident CAV1 (and perhaps other proteins such as flotillin) could act as an essential scaffold for organizing and ordering cholesterol molecules at MAMs, conferring them with a particular spatial arrangement, protein composition, and structural stability. A third, non-exclusive hypothesis could be that ER-mitochondria contacts through MAMs have functions beneficial for healthy cells, but detrimental in pathological situations- so MAM receding could be part of unspecific, protective mechanisms, engaged as part of organelle homeostatic programs, so far poorly characterized^19^. This third interpretation might be compatible with the fact that CAV1 deficiency leads to substantial alterations of the architecture of the ER and the mitochondria. However, functionally unrelated proteins, including well-established MAM components such as mitofusin 2, lead to similar structural changes of these organelles, and we cannot establish at this point a cause-consequence relationship between organelle architecture and MAM integrity.

Our unprecedented quantitative proteomics analysis yields interesting insights regarding functions that are accrued at MAMs and are significantly impacted by CAV1 deficiency (Fig. 3). The most prominent functional group is composed by regulators of steroid biosynthesis and cholesterol metabolism- an obvious functional branch affected by CAV1 dysfunction. Interestingly, other hits could be linked to steroid metabolism homeostasis through manual curation of their annotated functional categories- a very interesting example are several members of the Major Urinary Protein family (Mup 3, 8, 11 and 20). These proteins are commonly described as liver-produced feromones, dependent on steroid hormone signaling- but two studies also link their intracellular levels to mitochondrial energy management and performance^20^.

In humans, CAV1 mutations result in lipodystrophies and CAV1KO display a phenotype of partial lipodystrophy and resistance to obesity. In the liver, CAV1KO show an important intracellular lipid imbalance, decreased formation of lipid droplets and a cholesterol-promoted mitochondrial dysfunction^6^,^10^. Consistently, we found that the levels of cholesterol in MAM were significantly increased in CAV1KO, supporting the notion that CAV1 is involved in intracellular cholesterol regulation^6^. A minor yet interesting feature of CAV1KO is their impaired VLDL secretion^10^. Of note, we found that apolipoprotein E was decreased in the CAV1KO MAM preparation compared to WT. This protein is required for lipid mobilization and recruitment during VLDL assembly and trafficking through the ER membrane^21^. We therefore hypothesize that lack of apolipoprotein E in MAM might hamper the lipidation of nascent VLDL, contributing to a reduced VLDL secretion. Such disturbance in intracellular lipoprotein traffic in CAV1KO, to be confirmed in future research, would be consistent with previous results reporting impaired cholesterol exit from ER to Golgi in cells lacking functional CAV1^5^,^6^. Apolipoprotein E variants can also significantly affect MAM integrity and function in *in vitro* models^22^, and thus altered Apolipoprotein E recruitment to MAMs could be both a consequence of CAV1 deficiency and a cause for subsequent structural alterations of this system.

All in all, in this work we report a comprehensive list of proteins of WT hepatic MAMs as assessed by proteomics and show that CAV1 is a MAM-resident protein. We additionally explore the role of CAV1 on MAM structure, and describe a list of hepatic proteins either increased or decreased as a result of lack of functional CAV1, revealing a possible mechanistic link that explains why VLDL synthesis is impaired in CAV1KO mice. Future studies will further deepen on the mechanistic link between CAV1-dependent functions and MAM integrity, and the relevance of this endomembrane subdomain in CAV1 biology and disease.

## Methods

### Animals and isolation of cell fractions

All animal procedures conformed to EU Directive 86/609/EEC and Recommendation 2007/526/EC regarding the protection of animals used for experimental and other scientific purposes, enforced in Spanish law under Real Decreto 1201/2005. The experimental protocol was approved by the Carlos III National Centre for Cardiovascular Research (CNIC) Foundation Ethics Committee. 12 week-old male C57BL/6 J WT and CAV1KO were kept under a controlled humidity and lighting schedule with a 12-h dark period. Animals were starved overnight, killed by decapitation and livers were obtained. An aliquot of liver homogenate was immediately frozen, and MAMs, mitochondria, and ER were isolated following the protocol of Wieckowski and co-workers^8^ with slight modifications. While the original paper^8^ reports the separation of two bands corresponding to unpurified MAMs and unpurified mitochondria (“step 4”), in our case these bands remained attached. They were collected and were separated by different ultracentrifugation steps, rendering two fractions corresponding to purified mitochondria and pure MAMs, respectively (see Supplementary Fig. 1).

### Immunological techniques

Homogenate, purified ER, purified MAMs and purified mitochondria (50 μg of protein in all cases) were loaded on 12% sodium dodecyl sulfate-polyacrylamide gel electrophoresis (SDS-PAGE) (BioRad) and transferred onto nitrocellulose membranes (GE Lifesciences). Proteins were detected with the following antibodies: calreticulin, CAV1, and GM130 (BD Bioscience); TOMM20, cytochrome C, and COXI (Mitosciences); ACSL1, ACSL3, and ACSL4/FACL4 (Abnova); and Annexin A6 and ALDI, as described^23^. Secondary antibodies used were peroxidase-conjugated anti-mouse or anti-rabbit (Invitrogen). Signal was detected using the Enhance Chemiluminescence Detection kit (GE Healthcare).

To immunoprecipitate CAV1, MAMs from WT and CAV1KO were resuspended in 1 ml of immunoprecipitation buffer (10 mM Tris, 150 mM NaCl, 1% Triton X-100, SDS 0.1%, 0.1% deoxycholate, pH 7.35) in the presence of protease inhibitors. Antibody-protein binding was facilitated by the addition of a 1:100 dilution of the anti-CAV1. The mixtures were incubated for 3 h at 4 °C and then 50 μl of a 1:1 suspension of Sepharose:Protein A-Sepharose beads (Amersham Pharmacia Biotech) was added to each sample and the incubation was maintained for 2 h at 4 °C. Immunoprecipitates were centrifuged and the supernatant was removed. Immunoprecipitates were then washed three times in 0.5 ml of ice-cold PBS and proteins were digested using the in-gel digestion protocol as previously described^24^, with some modifications. Briefly, the samples were run by conventional SDS-PAGE until the front entered 3 mm into the resolving gel. The protein band containing the whole proteome was visualized by Coomassie staining, excised, cut into cubes, subjected to reduction with 10 mM DTT and alkylation in 50 mM iodoacetamide, and digested overnight at 37 °C with 60 ng/ml modified trypsin (Promega) at 12:1 protein:trypsin (w/w) ratio in 50 mM ammonium bicarbonate, pH 8.8 containing 10% acetonitrile. The resulting tryptic peptides were extracted by incubation in 12 mM ammonium bicarbonate pH 8.8 and later, 0.5% TFA. TFA was added to a final concentration of 1% and the peptides were finally desalted onto C18 Oasis-HLB cartridges and dried-down for mass spectrometry analysis.

### Proteomics analysis

#### Protein preparation

MAM proteins were digested using the filter aided sample preparation (FASP) protocol^25^. Briefly, samples were dissolved in 50 mM Tris-HCl pH 8.5, 4% SDS and 50 mM DTT, boiled for 10 min and centrifuged. Protein concentration in the supernatant was measured by the Direct Detect^®^ Spectrometer (Millipore). About 100 μg of protein were diluted in 8 M urea in 0.1 M Tris-HCl pH 8.5 (UA), and loaded onto 30 kDa centrifugal filter devices (FASP Protein Digestion Kit). The denaturation buffer was replaced by washing three times with UA. Proteins were then alkylated using 50 mM iodoacetamide in UA for 20 min in the dark, and the excess of alkylation reagents was eliminated by washing three times with UA and three additional times with 50 mM ammonium bicarbonate. Proteins were digested overnight at 37 °C with modified trypsin (Promega) in 50 mM ammonium bicarbonate at 50:1 protein:trypsin (w/w) ratio. The resulting peptides were eluted by centrifugation with 50 mM ammonium bicarbonate (twice) and 0.5 M sodium chloride. Trifluoroacetic acid (TFA) was added to a final concentration of 1% and the peptides were finally desalted onto C18 Oasis-HLB cartridges and dried-down for further analysis.

#### Stable isobaric labeling

For the quantitative analysis, tryptic peptides were dissolved in Triethylammonium bicarbonate (TEAB) buffer, and the concentration of peptides was determined by measuring amide bonds with the Direct Detect system. Equal amounts of each peptide sample were labeled using the 4-plex iTRAQ Reagents Multiplex Kit (Applied Biosystems) according to manufacturer’s protocol. Briefly, each peptide solution was independently labeled at room temperature for 1 h with one iTRAQ reagent vial (mass tag 114, 115, 116 or 117) previously reconstituted with ethanol. After incubation at room temperature for 1 h, reaction was stopped with diluted TFA and peptides were combined. Samples were concentrated in a Speed Vac, desalted onto C18 Oasis-HLB cartridges and dried-down for mass spectrometry analysis.

#### Mass spectrometry

Digested peptides were loaded into the LC-MS/MS system for on-line desalting onto C18 cartridges and analyzing by LC-MS/MS using a C-18 reversed phase nano-column (75 μm I.D. ×25 cm, 2 μm particle size, Acclaim PepMap RSLC, 100 C18; Thermo Fisher Scientific) in a continuous acetonitrile gradient consisting of 0–30% B in 240 min, 50–90% B in 3 min (A = 0.1% formic acid; B = 90% acetonitrile, 0.1% formic acid). A flow rate of 200 nl/min was used to elute peptides from the RP nano-column to an emitter nanospray needle for real time ionization and peptide fragmentation on either a Orbitrap Elite mass spectrometer (Thermo Fisher), for label-free analysis or a QExactive, for iTRAQ-labeled samples. An enhanced FT-resolution spectrum (resolution = 70.000) followed by the CID MS/MS spectra from the 12 most intense parent ions were analyzed along the chromatographic run. Dynamic exclusion was set at 30 s. For increasing proteome coverage, labeled samples were also fractionated by cation exchange chromatography (Oasis HLB-MCX columns) into four fractions, which were desalted and analyzed by using the same system and conditions described before.

#### Protein identification and quantification

All spectra were analyzed with Proteome Discoverer (version 1.4.0.29, Thermo Fisher Scientific) using SEQUEST-HT (Thermo Fisher Scientific). For database searching at the Uniprot database containing all sequences from mouse (March 03, 2013), search parameters were selected as follows: trypsin digestion with 2 maximum missed cleavage sites, precursor and fragment mass tolerances of 600 ppm and 1.2 Da, respectively for Orbitrap Elite or 2 Da and 0.02 Da, respectively for QExactive (iTRAQ-labeled samples), carbamidomethyl cysteine as fixed modification and methionine oxidation as dynamic modifications. For iTRAQ labeled peptides, N-terminal and Lys iTRAQ modifications were selected as a fixed modification. Peptide identification was validated using the probability ratio method^26^ with an additional filtering for a precursor mass tolerance of 12 ppm. False discovery rate (FDR) was calculated using inverted databases and the refined method^27^ was used to filter peptides for quantitation, as previously described^24^. Protein quantification from reporter ion intensities and statistical analysis of quantitative data were performed using QuiXoT, based on a statistical model previously described^28^,^29^. In this model protein log2-ratios are expressed as a standardized variable, i.e., in units of standard deviation according to their estimated variances (Zq values). For functional protein analysis, proteins were classified in terms of the Gene Ontology Biological Process and Cellular Component, KEGG, IPA, and DAVID and analysis of altered categories was analyzed as described^28^.

### Electron microscopy

Liver from WT and CAV1KO (n = 1 each) was processed for electron microscopy using a modification of the method of Nguyen *et al*.^29^, as follows. Pieces of liver were rapidly excised and immersed in a solution of 2.5% glutaraldehyde in PBS and immediately irradiated in a Pelco Biowave (Ted Pella In) for 3 min at 80 watt under vacuum. Samples were transferred to a fresh solution of 2.5% glutaraldehyde in PBS and left for 30 min at room temperature before rinsing 4 × 2 min in 0.1 M cacodylate buffer. Samples were then immersed in a solution containing potassium ferricyanide (3%) and osmium tetroxide (2%) in 0.1 M cacodylate buffer for 30 min at room temperature. Following 6 × 3 min washes in distilled water the tissue blocks were then incubated in a filtered solution containing thiocarbohydrazide (1%) for 30 min at room temperature. After subsequent washing in distilled water (6 × 2 min) samples were incubated in an aqueous solution of osmium tetroxide (2%) for 30 min, then in distilled water (6 × 2 min) and incubated in 1% aqueous uranyl acetate for 30 min at 4 °C. After further distilled water washes (2 × 2 min) a freshly prepared filtered solution of 0.06% lead nitrate in aspartic acid (pH 5.5) warmed to 60 °C was added to the dish and further incubated for 20 min at 60 °C before rinsing in distilled water (6 × 3 min) at room temperature. Tissue blocks were dehydrated twice in each ethanol solution of 30%, 50%, 70%, 90% and absolute ethanol for 40 sec at 250 watt in the Pelco Biowave. Epon LX112 resin was used for embedding with infiltration steps at 25%, 50%, and 75% resin:absolute ethanol in the Pelco Biowave under vacuum at 250 watt for 3 min and finishing with 100% resin (twice), before the final embedding/blocking and curing at 60 °C for 12 h. A set of over 20 random images from each liver were taken at a magnification of 20kx. ER-mitochondria contacts were manually traced and quantified by two expert observers (C.F. and R.G.P.).

### Visualization of ER-mitochondrial contact by FRET measurements

Rapamycin-inducible fluorescent interorganelle linkers were used to study ER-mitochondrial interactions in 104 cells and three independent experiments. WT and CAV1KO embryonic fibroblasts were cotransfected with two constructs, kindly provided by Dr G. Hajnóczky, that directly target the cytoplasmic surface of the ER (CFP-FRB-ER) and the cytoplasmic surface of the mitochondria (AKAP-FKBP-YFP)^12^. Addition of rapamycin causes rapid heterodymerization between neighboring FKBP and FRB domains inducing tethering of membranes from two organelles. The interaction was assessed by flow cytometry-based FRET assay^30^ using a FACSCantoII (BD Biosciences) equipped with a 405, 488, and 635 nm lasers. To measure CFP and FRET cells were excited with the 405 nm laser and CFP signal was collected with a 450/50 filter, while the FRET-signal was collected with a 525/50 filter. To measure YFP, cells were excited with the 488 nm laser and the emission was measured with the 530/30 filter.

### Cholesterol measurement

The amount of cholesterol in MAMs was determined using the Amplex^TM^ Red Cholesterol Assay Kit (Molecular Probes) according to manufacturer’s instructions.

### Assessment of relative mitochondrial mass

WT or CAV1KO mouse embryonic fibroblasts (MEFs) were grown to 85% confluency, pulse labelled with 50 nM MitoTracker DeepRed (MolecularProbes) for 20 min, harvested and washed in warm PBS, and fixed for 10 min in 1% paraformaldehyde, pH 7.8. Relative mitochondrial content was estimated from accumulated signal amplitude from 50000 gated, single cells per condition in triplicate. Data were analyzed with the FlowJo 7.2 platform.

### Assessment of the degree of mitochondrial fragmentation

WT and CAV1KO (MEFs and normal epithelial breast human cells, MCF10A) were compared for the estimated degree of mitochondrial network fragmentation using both quantitative image analysis using the Opera automated microscope and the Acapella Studio software package, and qualitative image acquisition at superresolution using an SP8 Leica station.

Quantitative image analysis was performed on six biological replicates per condition, each comprising an average 2000 cells. Briefly, cells were grown to a confluency of approx. 60%, pulse-labelled with 50 nM MitoTracker DeepRed for 20 min at 37 °C, and immediately fixed at 4% paraformaldehyde for a further 20 min. After counterstaining with cytoplasmic (RpS11) and nuclear (Hoeschtt 33342) markers following standard immunofluorescence procedures, cells were imaged in a spinning disk Opera confocal microscopy with a 40X water immersion objective. Image analysis proceedings comprised (i) nuclear segmentation; (ii) cytoplasm segmentation; (iii) exclusion of out-of-focus cells and mitotic cells based on morphological, contrast and intensity properties; (iv) subsegmentation of the mitochondrial area and its overlap with the peripheral cell area; and (v) subobject (mitochondrial fragment) segmentation and size calculation. Approx. 35000 total mitochondrial fragments were assessed per condition, and their average distribution in five size classes across replicates was calculated.

Superresolution image acquisition was performed on an SP8 Leica laser scanning confocal microscope equipped with two STED laser lines at 562 and 660 nm. WT and CAV1KO cell samples were processed as recommended by standard STED guidelines and double-labelled with MitoTracker Orange and an anti-calreticulin antibody (Abcam) detected with an Alexa488 anti-rabbit conjugate (MolecularProbes). Z-stacks were acquired with an 80 nm-step using a 100X oil immersion objective and HyPer photodetectors. Image processing and visualization was performed using the Imaris software package.

### Functional enrichment analysis of differential mam components

A hit list of 51 proteins with a |log2 ratio| > 1.5 when comparing both replicate sets between wild type samples and CAV1KO samples was queried in the functional annotation engine DAVID (https://david.ncifcrf.gov/31), using as a tailored background list our proprietary database of wild type MAM components (Supplementary File 2). Proteins not included automatically in functional classes with an enrichment above *p* < 10^−2^ were further manually queried for related functions not automatically detected.

### Statistics

The statistical significance of differences was determined using the Student’s t test; *P < 0.05, ***P < 0.01.

## Additional Information

**How to cite this article**: Sala-Vila, A. *et al*. Interplay between hepatic mitochondria-associated membranes, lipid metabolism and caveolin-1 in mice. *Sci. Rep.* **6**, 27351; doi: 10.1038/srep27351 (2016).

## Supplementary Material

Supplementary Information

Supplementary Dataset 1

Supplementary Dataset 2
