# Supplementary material for: Interplay between hepatic mitochondria-associated membranes, lipid metabolism and caveolin-1 in mice
Source: Sci Rep. 2016 Jun 6;6:27351. doi: 10.1038/srep27351 (PMC4894368; doi:10.1038/srep27351)
Supplement: Supplementary Information [file srep27351-s1.pdf]

Supplemental material for "**Interplay between hepatic mitochondria-associated membranes, lipid metabolism and caveolin-1 in mice**", by Aleix-Sala Vila, Inmaculada Navarro-Lérida, Miguel Sánchez-Alvarez, Marta Bosch, Carlos Calvo, Juan Antonio López, Enrique Calvo, Charles Ferguson, Marta Giacomello, Annalisa Serafini, Luca Scorrano, José Antonio Enriquez, Jesús Balsinde, Robert G. Parton, Jesús Vázquez, Albert Pol, and Miguel Ángel del Pozo.

# SUPPLEMENTARY FIGURE 1

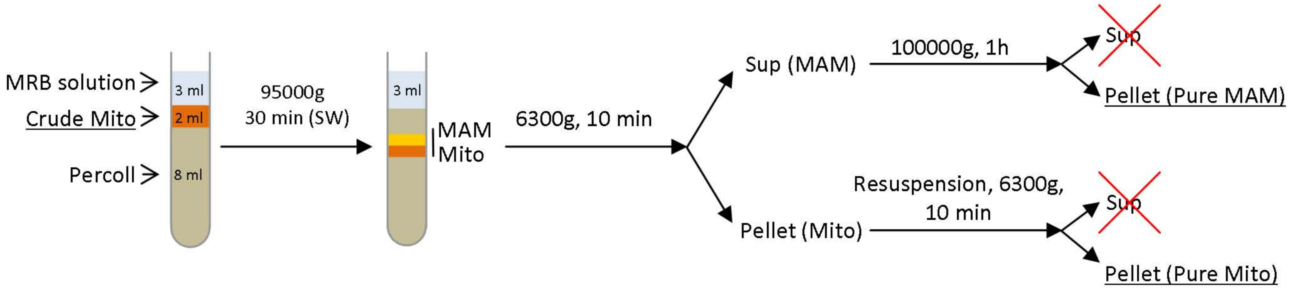


**Supplementary Figure 1**. Scheme of MAM and mitochondria (“Mito”) purification from crude mitochondria extract (“crude mito”) (adapted from Wieckowski MR et al. Nat Protoc 2009; 4: 1582–1590).

SUPPLEMENTARY FIGURE 2

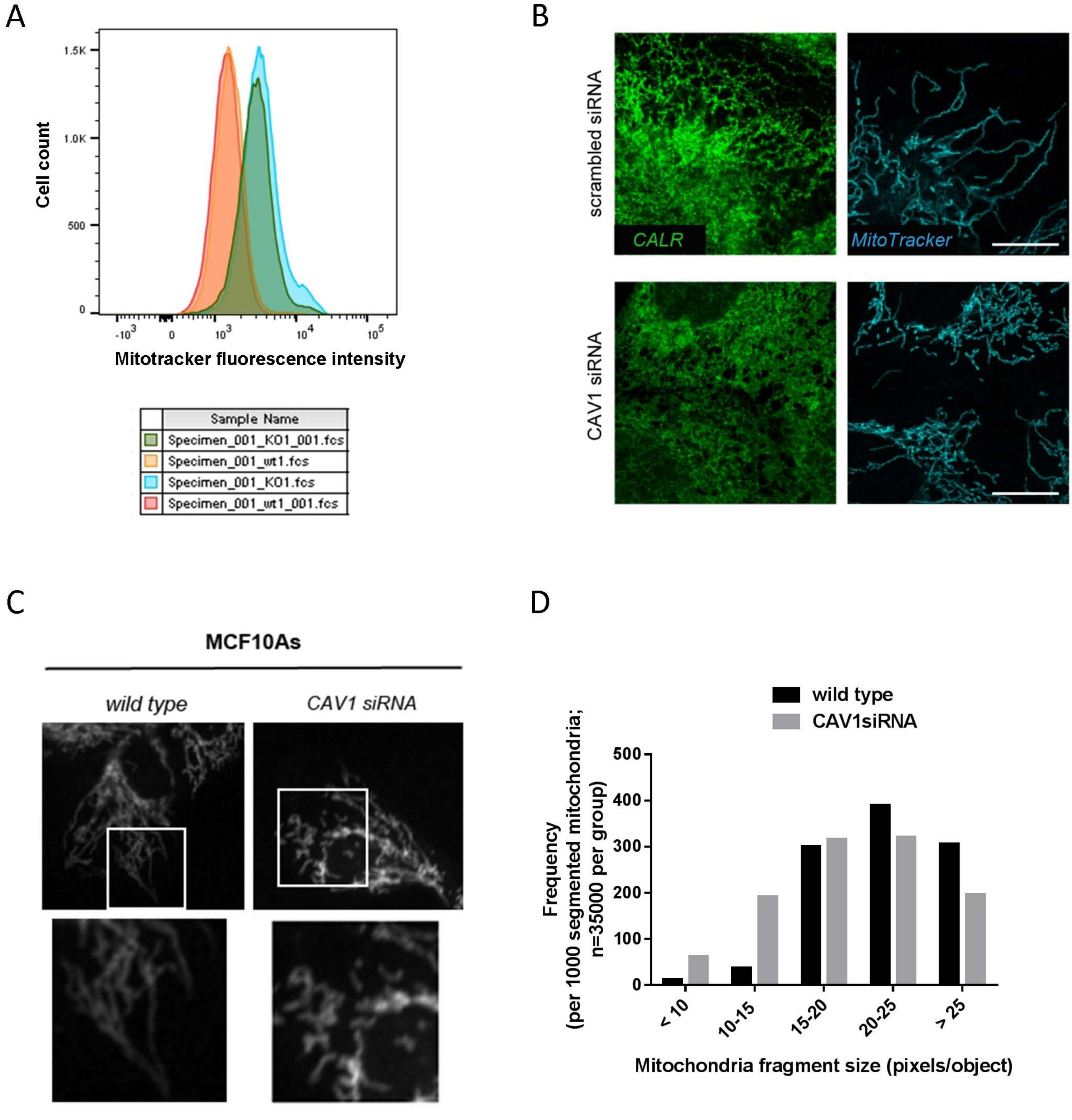


**Supplementary Figure 2**.
(A) Determination of relative mitochondrial mass by flow cytometry.
(B) STED optical sections of MCF10A cells transiently transfected with the indicated siRNAs. Cells were loaded with MitoTracker FarRed and processed for immunofluorescence staining using an anti-calreticulin antibody. White bar: 5 microns.
(C) Representative spinning disk confocal image of wild-type and CAV1-depleted MCF10A cell line after Mitotracker staining. Lower panels show enlarged views of mitochondria network.
(D) Quantitation of mitochondrial size distribution in wild type MCF10A cells as compared with CAV1 KD cells. n=35000 segmented mitochondrial bodies per background.
